# Pediatric liver transplant outcomes: A comparative analysis of steatotic donor grafts

**DOI:** 10.1002/jpn3.70213

**Published:** 2025-09-22

**Authors:** Ahmad Anouti, Hamza Dahshi, Madhukar S. Patel, Thomas G. Cotter, Julie K. Heimbach, Sara Hassan

**Affiliations:** ^1^ Department of Pediatrics University of Texas Southwestern Medical Center Dallas Texas USA; ^2^ School of Medicine Case Western Reserve University Cleveland Ohio USA; ^3^ Department of Surgery University of Texas Southwestern Medical Center Dallas Texas USA; ^4^ Division of Digestive and Liver Diseases University of Texas Southwestern Medical Center Dallas Texas USA; ^5^ Department of Surgery Mayo Clinic Rochester Minnesota USA; ^6^ Department of Pediatrics, Pediatric Gastroenterology, Hepatology, and Nutrition University of Texas Southwestern Medical Center Dallas Texas USA

**Keywords:** donor graft steatosis, liver transplant, steatotic graft outcomes

## Abstract

**Objectives:**

Hepatic steatosis impacts the quality of grafts, affecting transplant outcomes. Rising obesity rates and subsequent donor graft steatosis further influence the organ shortage crisis in pediatric liver transplantation (LT). Our study aimed to evaluate how donor steatosis modulates the outcomes of pediatric LT.

**Methods:**

We analyzed the United Network of Organ Sharing database for transplanted donor grafts from January 01, 2004, to April 30, 2024. We stratified pediatric (≤18 years) LT recipients into steatotic grafts, subdivided into <30% and ≥30%. Graft failure was assessed using Kaplan–Meier curves, and Cox proportional hazards models, with Lasso regression identifying key predictive variables. Gradient‐boosting decision tree was used to assess the level of likelihood importance for post‐LT survival.

**Results:**

Five hundred and ninety‐five pediatric LT recipients were included; 62 (10.4%) received donors with steatosis levels ≥30%. Survival rates for steatotic grafts ≥30% were 93.5% at 1 year, 89.9% at 5 years, and 84.4% at 10 years, compared to 94.7%, 89.5%, and 85.2% respectively, among steatotic grafts <30% (*p* = 0.72, *p* = 0.92, and *p* = 0.92). Donor age (adjusted hazard ratio [aHR]: 1.01, 95% confidence interval [CI]: 1.01–1.03), donation after cardiac death (DCD) (aHR: 10.68, 95% CI: 3.27–34.86), and recipient life support (aHR: 1.95, 95% CI: 1.19–3.20) were associated with an increased risk of mortality.

**Conclusion:**

Steatotic grafts in pediatric patients had acceptable outcomes. Predictors of mortality in steatotic grafts, including donor age, DCD, and recipient life support, underscore the complex interplay of multiple factors in post‐LT outcomes.

## Abstract (highlights)

**What is Known:**

- There is a shortage of liver grafts available for transplantation.
- Obesity is on the rise thus resulting in an increased rate of steatotic grafts.
- Adult recipients of steatotic grafts have been shown to have worse outcomes.

**What is New:**

- Pediatric patients are receiving steatotic liver grafts for transplant.
- Unlike adult recipients, pediatric patients have excellent outcomes comparable to those of none steatotic grafts.
- Likelihood of predicting mortality in pediatric patients with steatotic graftscan be assessed using specific variables such as age and life support requirements.

## Graphical abstract

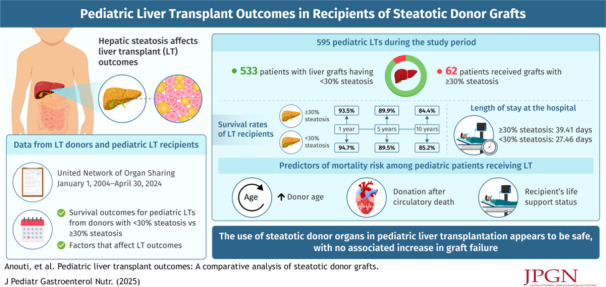

## 1 INTRODUCTION

The increasing prevalence of obesity worldwide has affected the quality of available livers for donation.^1^ Obesity often leads to metabolic dysfunction associated with steatotic liver disease, which can lead to an increase in hepatic steatosis in potential donor organs.^2^ Steatosis, the pathological accumulation of fat within liver cells, is categorized based on the size and distribution of fat in the cytoplasm of hepatocytes. Microvesicular steatosis involves the accumulation of numerous small lipid droplets that do not displace the hepatocyte nucleus, whereas macrovesicular steatosis is characterized by larger fat droplets that push the nucleus to the periphery, potentially compromising liver function.^3^ Generally, livers with 30% or more steatosis are considered less desirable for liver transplant (LT) owing to the higher risks of poor outcomes post‐transplant, including graft failure and higher mortality rates.^4^

There remains an organ shortage affecting pediatric LT recipients. In 2022, there were 526 pediatric LT candidates, with 741 new patients on the waitlist.^5^ Despite innovation in surgical techniques and use of technical variant grafts, an ongoing need remains to increase organ availability. Given the increasing prevalence of obesity among the donor population, along with a shortage of organs available for pediatric LT recipients, our study aimed to provide a detailed evaluation of the outcomes for pediatric LT recipients who have received livers with significant levels of steatosis ≥30%. Our secondary aims were to (1) explore the role of steatosis in pediatric LT and (2) identify the key factors that influence LT outcomes in this high‐risk pediatric population.

## 2 METHODS

### 2.1 Ethics statement

Our study was exempted by the UT Southwestern Medical Center Institutional Review Board (IRB). The UT Southwestern Medical Center Institutional Review Board (IRB) considered this study as an example.

### 2.2 Study population and characteristics

Patients were identified using the Organ Procurement Transplantation Network (OPTN)/United Network of Organ Sharing (UNOS) Standard Transplant Analysis and Research (STAR) file (created on April 30, 2024). Data from LT donors and pediatric LT recipients (age ≤18 years) from January 1, 2004, to April 30, 2024, were analyzed. The inclusion criteria were LT donors with documented steatosis levels and/or transplant recipients of these organs.

Donor characteristics included age, ethnicity, sex, body mass index (BMI), split or partial donation, UNOS region, ABO blood type, laboratory values (total bilirubin, alanine transaminase, aspartate transaminase, and creatinine), BMI (kg/m^2^), weight (kg), microvesicular and macrovesicular steatosis (<30% or ≥30%), donation after cardiac death (DCD), donor liver biopsy, and donor liver fibrosis. Only donor allografts with biopsy‐proven steatosis were included in the study. Clinical data for LT recipients included age, sex, race, ethnicity, BMI, cold ischemia time (CIT), Pediatric end‐stage liver disease (PELD)‐Na score, presence of encephalopathy (yes/no), ascites (yes/no), comorbidities, laboratory values (total bilirubin, albumin, and creatinine), BMI (kg/m^2^), weight (kg) time on the waitlist, insurance type, days on the waitlist, length of hospital stay, intensive care unit (ICU) stay, UNOS region, and education level.

### 2.3 Statistical analysis

The primary comparative analyses compared patients with any form of steatosis (macrovesicular, microvesicular, or both) <30% to those with steatosis ≥30%. Furthermore, a sensitivity analysis was performed to further stratify microvesicular and macrovesicular steatosis. The sensitivity analysis was compromised of four groups based on the presence and extent of macrovesicular and microvesicular steatosis: macrovesicular <30%, macrovesicular ≥30%, microvesicular <30%, and microvesicular ≥30%. A single graft could be classified into both macrovesicular and microvesicular categories. The primary outcome was graft failure in LT recipients. Associations with steatotic graft outcomes among the LT recipients were also evaluated.

Continuous variables are summarized as medians and interquartile ranges (IQR), and frequencies and percentages are used for categorical variables. Comparative analysis of continuous variables was based on a two‐sample Wilcoxon rank test for samples that failed the Shapiro–Wilk normality test. Otherwise, it was based on a two‐sample Wilcoxon rank test for samples that failed the Shapiro–Wilk normality test. Otherwise, a two‐sample *t*‐test was used. A comparative analysis of categorical variables was performed using a two‐sided chi‐squared test. Analyses were adjusted a priori for clinically significant variables, including recipient age, CIT, donor age, diabetes, recipient BMI, physiological PELD score, life support at the time of transplant, UNOS region, and status 1 A/B.

As per the OPTN, graft survival was defined as the absence of either recipient death or re‐transplantation. LT graft survival was estimated using Kaplan–Meier analyses and comparisons between the LT recipient groups. Adjusted *p*‐values were obtained via multivariable Cox proportional hazards analysis using variables, selected a priori, which have been associated with graft failure (i.e., donor variables: age, history of diabetes, heavy alcohol use, graft macrovesicular steatosis <30%, graft microvesicular steatosis <30%, donation after circulatory death (DCD); recipient variables: age, diabetes, life support, status 1 A/B, ICU stay, PELD, center volumes, transplant era, and UNOS region).^6^, ^7^, ^8^, ^9^ Model violations and assumptions were assessed, including multicollinearity and proportional hazards.

Associations with graft outcomes (i.e., graft failure) among LT recipients were assessed using Cox proportional hazards modeling with the incorporation of variables such as donor: age, history of diabetes, heavy alcohol use, graft macrovesicular steatosis <30%, graft microvesicular steatosis <30%, DCD; recipient: age, diabetes, life support, status 1 A/B, ICU stay, PELD, center volumes, transplant era, and UNOS regions.

The Least Absolute Shrinkage and Selection Operator (LASSO) variable selection, a machine learning technique, was applied to identify key predictive variables of the Cox proportional hazards model, assessing variables associated with outcomes. Lasso achieved this by introducing a regularization penalty proportional to the absolute value of the regression coefficients. This penalty helps reduce overfitting by shrinking the less significant coefficients to zero, thus performing variable selection automatically. We then reran the Cox proportional hazards model using the variables selected by LASSO to obtain more accurate and adjusted hazard ratio values.

On the sensitivity analysis, we complemented the Lasso Model with Extreme Gradient Boosting (XGBoost), a machine‐learning algorithm based on optimized distributed gradient boosting decision trees, to assess the level of likelihood importance in graft outcomes for each variable.

Statistical analyses were completed using R and R Studio, and *p*‐values < 0.05 were considered significant for all analyses.

## 3 RESULTS

### 3.1 Study population characteristics

**Figure 1:**
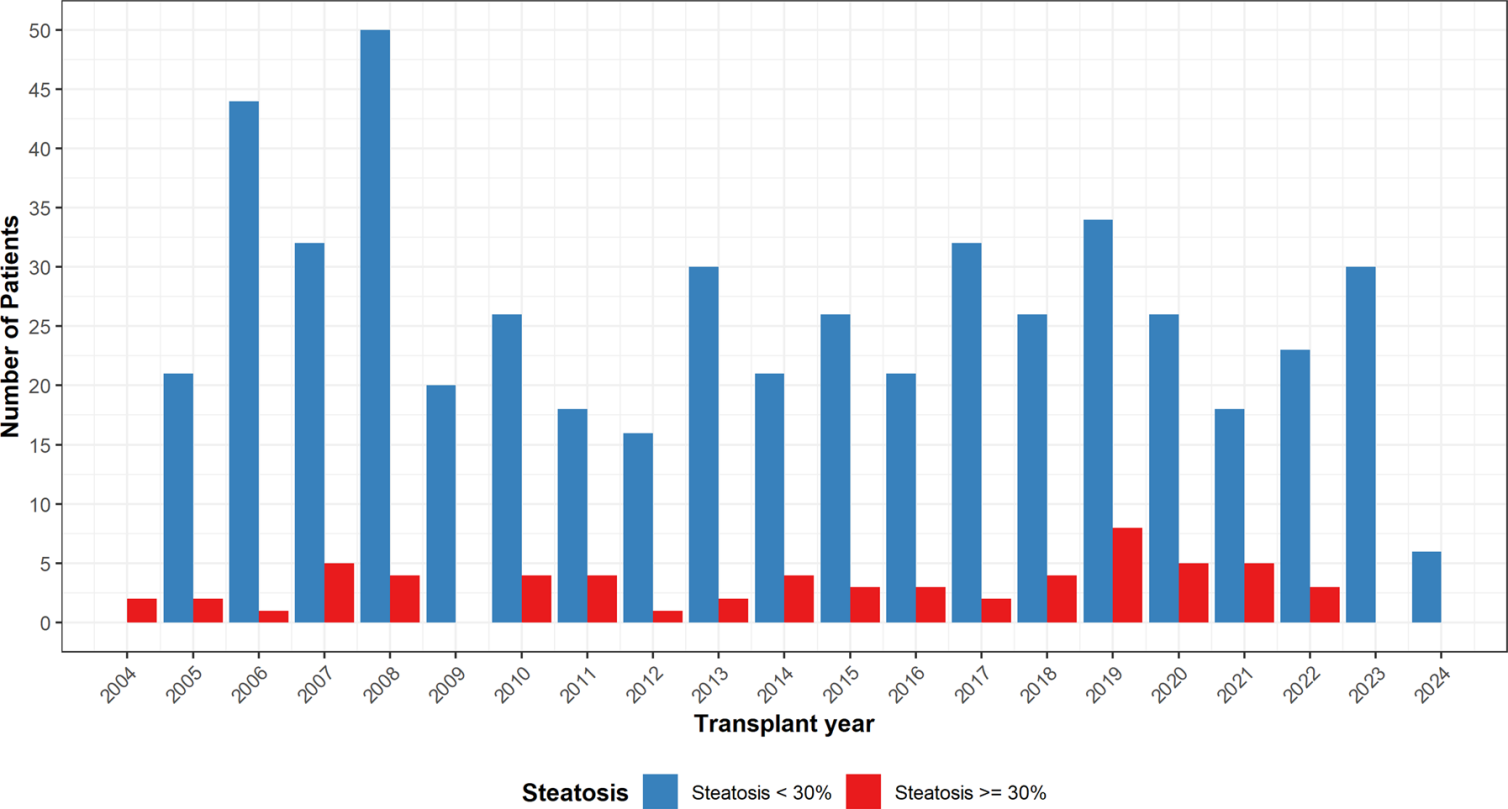
Distribution of steatotic donated livers to pediatric transplant recipients by stratified by year.

During the study period, 10,244 pediatric LTs were performed, of which 595 (5.8%) pediatric LT recipients aged ≤18 years had data recorded on either macrovesicular steatosis, microvesicular steatosis, or both. A total of 533 (89.6%) pediatric LT recipients received donors with steatosis <30% and 62 (10.4%) received grafts with steatosis ≥30%, respectively. The number of pediatric LT recipients with grafts with steatosis ≥30% has remained sparse and stable over time (Figure 1). Among those with steatosis <30%, the most common indication for transplantation was biliary atresia (BA) at 24.2% (*n* = 144). Similarly, the most common indications for transplantation among the steatosis ≥30% groups were BA with 15 (24.2%) patients.

In examining the characteristics between donors with steatosis <30% and those with ≥30%, history of hypertension was significantly higher in donors with steatosis <30%, with 66 cases (12.4%) compared to seven cases (11.3%) in those with steatosis ≥30% (*p* = 0.01). Additionally, liver fibrosis was more prevalent in donors with steatosis ≥30%, where it affected seven individuals (11.3%), as opposed to 19 individuals (3.6%) in the <30% group (*p* = 0.04). Finally, there was a significant variation in the distribution of steatotic donors based on UNOS region (*p* = 0.04). Recipient outcomes also had varying length of hospital stay; those receiving grafts from donors with higher steatosis had longer hospital stays, averaging 39.41 days compared to 27.46 days for those with lower steatosis (*p* = 0.04) (Table 1).

**Table 1:** All donor and recipient characteristics from 2004 to 2024 stratified by steatosis <30% and ≥30% and age ≤18 years old.

Variable	Steatosis <30% (*N* = 533)	Steatosis ≥30% (*N* = 62)	*p* value
Donor characteristics			
Age (years)	21.25 ± 17.63	21.24 ± 16.16	0.84
Female gender	240 (45.0%)	30 (48.4%)	0.71
Ethnicity			0.11
Non‐Hispanic White	332 (62.3%)	30 (48.4%)	
Non‐Hispanic Black	105 (19.7%)	12 (19.4%)	
Hispanic	79 (14.8%)	18 (29.0%)	
Asian	9 (1.7%)	2 (3.2%)	
American Indian/Alaskan Native	5 (0.9%)	0 (0%)	
Native Hawaiian/Other Pacific Islander	1 (0.2%)	0 (0%)	
Multiracial	2 (0.4%)	0 (0%)	
Diabetes history	27 (5.1%)	1 (1.6%)	0.72
Hypertension history	66 (12.4%)	7 (11.3%)	0.01^a^
Heavy alcohol use history (>2 drinks/day)	40 (7.5%)	5 (8.1%)	0.78
History of cigarette use	50 (9.4%)	3 (4.8%)	
History of HCV	2 (0.4%)	0 (0%)	0.82
History of HIV	0 (0%)	0 (0%)	0.99
Weight (kg)	52.17 ± 29.45	56.72 ± 27.85	0.30
Body mass index (kg/m^2^)	22.35 ± 6.00	23.55 ± 6.14	0.13
Last creatinine	1.22 ± 1.42	1.46 ± 1.84	0.17
Last total bilirubin	0.89 ± 0.88	1.11 ± 1.10	0.11
Last AST	109.23 ± 156.04	98.41 ± 116.18	0.91
Last ALT	95.92 ± 177.07	68.23 ± 78.05	0.87
Liver fibrosis			0.04^a^
Fibrosis (unspecified degree)	19 (3.6%)	7 (11.3%)	
Cirrhosis (incomplete and complete)	0 (0%)	0 (0%)	
Donation after cardiac donation	3 (0.6%)	1 (1.6%)	0.89
Blood type:			0.98
A	153 (28.7%)	20 (32.3%)	
B	48 (9.0%)	5 (8.1%)	
O	325 (61.0%)	37 (59.6%)	
AB	7 (1.3%)	0 (0%)	
UNOS region			0.04^a^
1	15 (2.8%)	3 (4.8%)	
2	32 (6.0%)	9 (14.5%)	
3	123 (23.1%)	16 (25.8%)	
4	23 (4.3%)	5 (8.1%)	
5	60 (11.3%)	7 (11.3%)	
6	27 (5.1%)	0 (0%)	
7	61 (11.4%)	4 (6.5%)	
8	70 (13.1%)	4 (6.5%)	
9	20 (3.8%)	3 (4.8%)	
10	41 (7.7%)	1 (1.6%)	
11	61 (11.4%)	10 (16.1%)	
Recipient characteristics			
Age (years)	8.11 ± 6.82	9.56 ± 6.60	0.16
Cold ischemia time (hours)	7.10 ± 2.94	7.03 ± 2.17	0.71
Transplant type			0.57
Split	391 (73.4%)	49 (79.0%)	
Whole	142 (26.6%)	13 (21.0%)	
Ethnicity			0.95
Non‐Hispanic White	272 (51.0%)	29 (46.8%)	
Non‐Hispanic Black	106 (19.9%)	13 (21.0%)	
Hispanic	108 (20.3%)	15 (24.2%)	
Asian	31 (5.8%)	4 (6.5%)	
Other	16 (3.0%)	1 (1.5%)	
Female gender	265 (49.7%)	32 (50.0%)	0.88
Diabetes	10 (1.9%)	2 (3.2%)	0.18
Body mass index (kg/m^2^)	19.49 ± 4.53	19.36 ± 4.16	0.90
Weight (kg)	33.8 ± 26.3	38.33 ± 24.69	0.15
Total bilirubin (mg/dL)	11.03 ± 11.70	12.67 ± 13.67	0.30
Albumin	3.20 ± 0.76	3.25 ± 0.64	0.50
Serum creatinine (mg/dL)	0.69 ± 1.02	0.85 ± 0.82	0.07
Length of stay (days)	27.46 ± 38.90	39.41 ± 51.97	0.04^a^
Days on waitlist	113.40 ± 227.85	149.58 ± 356.10	0.45
UNOS region			0.12
1	21 (3.9%)	3 (4.8%)	
2	47 (8.8%)	9 (14.5%)	
3	109 (20.5%)	10 (16.1%)	
4	26 (4.9%)	4 (6.5%)	
5	53 (9.9%)	6 (9.7%)	
6	22 (4.1%)	0 (0%)	
7	64 (12.0%)	6 (9.7%)	
8	81 (15.2%)	4 (6.5%)	
9	26 (4.9%)	8 (12.9%)	
10	28 (5.2%)	4 (6.5%)	
11	56 (10.5%)	8 (12.9%)	
Education level:			0.08
None	7 (1.3%)	0 (0%)	
Grade/high school	263 (49.3%)	35 (56.5%)	
College/Bachelor	9 (1.7%)	1 (1.6%)	
Postgraduate	0 (0%)	0 (0%)	
Insurance:			0.97
Private	243 (45.6%)	27(43.6%)	
Medicaid	228 (42.8%)	29 (46.8%)	
Medicare/CHIP	30 (5.6%)	3 (4.8%)	
VA/other gov.	13 (2.4%)	2 (2.3%)	
Self/donation	7 (1.3%)	0 (0%)	
Foreign	10 (1.9%)	1 (1.6%)	
Life support	108 (20.3%)	13 (21.0%)	0.99
Portal vein thrombosis	28 (5.3%)	5 (8.1%)	0.53
Intensive care unit	152 (28.5%)	20 (32.3%)	0.81
Ascites	308 (57.8%)	34 (54.8%)	0.61
Hepatic encephalopathy	265 (49.7%)	36 (58.1%)	0.35
Hepatocellular carcinoma	3 (0.6%)	1 (1.6%)	0.95
Dialysis	47 (8.8%)	6 (9.7%)	0.99
MELD/PELD score at transplant	17.73 ± 14.59	19.10 ± 13.86	0.47
MELD exception	197 (37.0%)	21 (33.9%)	0.48
Status 1A/B	194 (36.4%)	21 (33.9%)	0.80
Center volume:			0.12
Lower volume	288 (54.0%)	33 (53.2%)	
Medium volume	166 (31.1%)	25 (40.3%)	
High volume	79 (14.8%)	4 (6.5%)	
Transplant era:			0.17
2004–2009	180 (33.8%)	14 (22.6%)	
2010–2015	137 (25.7%)	18 (29.0%)	
2016–08/2021	148 (27.8%)	24 (38.7%)	
09/2021–2024	68 (12.8%)	6 (9.7%)	

Abbreviations: ALT, alanine aminotransferase; AST, aspartate aminotransferase; CHIP, Children's Health Insurance Program; HCV, hepatitis C virus; HIV, human immunodeficiency virus; MELD, model for end‐stage liver disease; PELD, pediatric end‐stage of liver disease; UNOS, United Network of Organ Sharing; VA, Veterans Affairs.

^a^ PELD and UNOS.

### 3.2 Recipient outcomes

**Figure 2:**
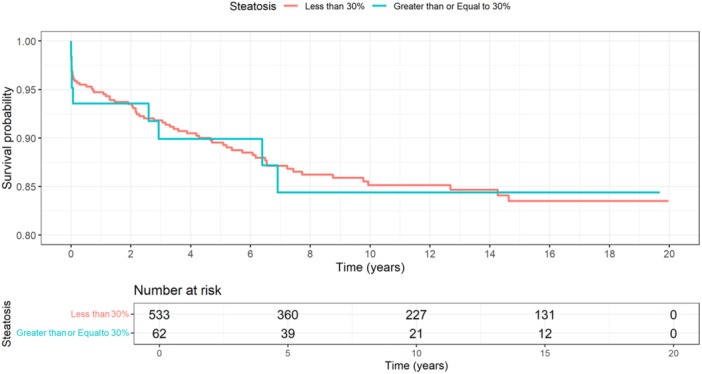
Survival analysis of pediatric LT recipients with with steatosis <30% and ≥30%. LT, liver transplantation.

Survival rates for steatotic grafts ≥30% were 93.5% at 1 year, 89.9% at 5 years, and 84.4% at 10 years, compared to 94.7%, 89.5%, and 85.2% respectively, among steatotic grafts <30% (*p* = 0.72, *p* = 0.92, and *p* = 0.92) (Figure 2). In the cox proportional hazard model only DCD grafts (adjusted hazard ratio [aHR]: 9.66, 95% confidence interval [CI]: 2.60–35.89) were found to be associated with an increased risk of mortality. However, on LASSO regression, donor age (aHR: 1.01, 95% CI: 1.01–1.03), DCD (aHR: 10.68, 95% CI: 3.27–34.86), and recipient life support (aHR: 1.95, 95% CI: 1.19–3.20) were associated with an increased risk of mortality. Steatosis ≥30% was not associated with a significant increase in mortality in either model (Table 2).

**Table 2:** Factors associated with mortality among mortality among the study population.

Variables	Cox proportional HR		Lasso regression	
	Adjusted HR (95% CI)	*p* value	Adjusted HR (95% CI)	*p* value
Donor age	1.01 (0.99–1.03)	0.15	1.01 (1.00–1.03)	0.03^a^
Donor history of diabetes	1.00 (0.34–2.99)	0.99	Dropped from lasso	
Donor heavy alcohol use	1.05 (0.47–2.38)	0.90	Dropped from lasso	
Steatosis ≥ 30%	0.98 (0.46–2.08)	0.95	Dropped from lasso	
DCD	9.66 (2.60–35.89)	<0.01^a^	10.68 (3.27–34.86)	<0.01^a^
Transplant type (ref: Whole)	1.05 (0.52–2.11)	0.89	Dropped from lasso	
Recipient age	1.01 (0.96–1.06)	0.77	Dropped from lasso	
Recipient diabetes	0.62 (0.08–4.61)	0.64	Dropped from lasso	
Recipient life support	2.09 (1.00–4.34)	0.049	1.95 (1.19–3.20)	0.01^a^
Recipient status 1 A/B	0.85 (0.42–1.71)	0.64	Dropped from lasso	
Recipient ICU stay	1.05 (0.44–2.48)	0.92	Dropped from lasso	
Recipient PELD	0.99 (0.98–1.02)	0.92	Dropped from lasso	
Center volume (Ref: Low vol)				
Middle volume	1.03 (0.57–1.84)	0.93	Dropped from lasso	
High volume	0.59 (0.25–1.39)	0.23	Dropped from lasso	
Transplant era (Ref: 2004–2009)				
2010–2015	0.90 (0.50–1.62)	0.72	Dropped from lasso	
2016–08/2021	0.72 (0.37–1.38)	0.32	Dropped from lasso	
09/2021–2024	0.20 (0.03–1.50)	0.12	0.22 (0.03–1.66)	0.14

*Note*: Dropped variables were variables that did not meet the lambda weight to be included in the final Lasso model. Abbreviations: CI, confidence interval; DCD, donation after cardiac death; HR, hazard ratios; ICU, intensive care unit; PELD, pediatric end‐stage of liver disease; UNOS, United Network for Organ Sharing.

^a^ Cox proportional model included and was adjusted for UNOS region.

### 3.3 Sensitivity analysis

Fourteen recipients (2.4%) and 53 recipients (9.4%) received donors with macrovesicular and microvesicular steatosis levels ≥30%, respectively (Table S1). For macrovesicular steatotic grafts ≥30%, the 1‐, 5‐, and 10‐year survival rates were 92.9%, 85.7%, and 85.7% respectively. In comparison, grafts with <30% macrovesicular steatosis had survival rates of 94.7%, 89.6%, and 84.9% at the same time points (*p* = 0.80, *p* = 0.68, and *p* = 0.68, respectively). For microvesicular steatotic grafts, the 1‐, 5‐, and 10‐year survival rates were 92.5%, 88.2%, and 82% for ≥30% microvesicular steatosis, and 95.1%, 89.7%, and 85% for <30% microvesicular steatosis (*p* = 0.49, *p* = 0.78, and *p* = 0.76, respectively) (Figure S1A,B). Sensitivity LASSO regression stratified by steatosis type showed, donor age (aHR: 1.01, 95% CI: 1.01–1.03), DCD (aHR: 9.35, 95% CI: 2.90–30.17), and recipient life support (aHR: 1.97, 95% CI: 1.19–3.26) were associated with increased mortality). Neither macrovesicular nor microvesicular steatosis were associated with increased mortality. The decision tree model stratified by steatosis type showed that Macrovesicular steatosis (0.4%) and microvesicular steatosis (2.8%) ≥30% were associated with a minimal likelihood of predicting survival in the study population (Table S2 and Figure S2). The variables included in the model (donor age, PELD score, recipient age, UNOS region, transplant era, ICU admission, recipient status 1 A/B, donor and recipient diabetes, donor heavy alcohol use, and recipient life support requirements) resulted in an area under the curve of 0.975, with a sensitivity of 97.1% and specificity of 92.1% in predicting the likelihood of survival among the study population.

## 4 DISCUSSION

With the increasing prevalence of steatotic donor grafts, there is a need to further understand the impact of steatosis on the pediatric population, which is currently challenged by organ shortage. Our study is the first to assess the impact of steatosis on the outcomes of pediatric LT recipients. Contrary to adult data implicating steatosis with poor posttransplant outcomes,^10^ our findings indicate that steatosis of ≥30%, whether macrovesicular or microvesicular, in donor livers does not seem to significantly affect the survival rates of carefully matched pediatric recipients within the study population. In contrast, specific variables such as the type of donated liver, recipient age, and PELD score played a significant role in defining graft failure in the steatotic study population.

Based on our sensitivity analyses, both macrovesicular and microvesicular steatotic grafts showed similar short‐ and long‐term survival rates, indicating that higher steatosis levels do not necessarily compromise pediatric LT recipient outcomes posttransplant. These results differ from those of studies that have shown worsened posttransplant survival outcomes in adult LT recipients with steatotic grafts.^11^, ^12^ De Graaf et al. demonstrated that adult recipients who received grafts with moderate (30%–60%) to severe (≥60%) macrovesicular steatosis experienced an increased risk of poor early allograft outcomes.^13^ In a recent study by Alvikas et al., these results were further solidified with grafts having moderate (>30%) macrovesicular steatosis have an 2.69 increased risk of mortality within 90‐days post‐LT.^14^ A recent study by Croome et al. showed that adult recipients of livers with significant microvesicular steatosis (≥30%) are at an increased risk of postreperfusion syndrome, early allograft dysfunction, and postoperative renal dysfunction requiring continuous renal replacement therapy.^10^ In addition to the poorer outcomes of steatotic LT grafts among adult recipients, pediatric LT recipients that received grafts with macrovesicular steatosis ≥30% have not been identified as a primary reason for graftrejection or failure.^15^ As reported by Guo et al., only a small proportion of biopsied donor grafts used in pediatric LTs (seven grafts, 14%) exhibited >30% macrovesicular steatosis, and this was not a significant factor contributing to graft failure.^15^

While steatosis did not significantly affect survival, certain variables were identified as increasing mortality risk among the study population. Specifically, older donor age, recipient life support status and DCD were associated with increased mortality risk. In a study by Xu et al., pediatric LT recipients who received split LTs from older donors had significantly lower survival rates compared to those receiving livers from younger donors.^16^, ^17^ Older donor age, along with increased steatosis, was also correlated with worse outcomes, as livers from older donors might have reduced regenerative capacity and increased vulnerability to ischemia‐reperfusion injury.^17^ Recipients with life support requirements are at an increased risk of mortality, which can be attributed to their more severe illness at the time of transplant and their heightened risk of complications.^18^, ^19^ The impact of these variables on mortality is specific to the study population and may not be generalizable to the broader pediatric LT population. DCD grafts were associated with a notably higher mortality risk within our study population. Worse outcomes in DCD are primarily due to severe graft injury caused by mandatory warm ischemia during organ recovery, which leads to increased risks of graft failure and ischemic biliary complications.^17^ DCD grafts are linked to higher incidences of primary nonfunction, ischemic cholangiopathy, vascular thrombosis, and posttransplant acute kidney injury due to the prolonged cold and warm ischemic times, which adversely affect graft viability.^20^, ^21^ In selecting steatotic grafts for pediatric LT, it may be prudent to review these factors associated with increased mortality.

Our study has limitations that should be acknowledged. The retrospective nature of the analysis and reliance on registry data introduces potential biases, including inaccuracies in data recording and unmeasured confounding factors. Additionally, the small sample size, particularly for recipients of livers with high steatosis levels, exposes our study to a type 2 error (i.e., failing to reject the null hypothesis when a true difference exists). The collective data represent a minority of the pediatric transplant population during the study period. Therefore, our results should be interpreted in this context. We mitigated the limitations of our small sample size by performing a robust analysis to help increase our confidence in the study findings. Another limitation of this study includes selection bias in the data. The significance of the UNOS region in comparisons across steatosis groups may suggest that some centers are more consistent than others in recording this data. Additionally, variation in surgical practices and the frequency of donor graft biopsies could further bias the results. Donor grafts selected for biopsy are selected due to the concern of possibly being lower quality or marginal grafts.^15^ These center‐specific practices may not only influence the recorded data but could also affect outcomes, as many are closely tied to specific centers. Future studies with larger sample sizes and prospective designs are required to validate our results. Furthermore, while our analysis adjusted for several key variables, other unaccounted factors may have influenced the outcomes. In the wake of the obesity epidemic and the rise in steatotic organs, future research should aim to incorporate a broader range of variables, including detailed recipient health status and perioperative management strategies.

## 5 CONCLUSION

Our findings suggest that the use of steatotic organs in pediatric LT recipients appears to be safe, with no increase in graft failure outcomes. Steatosis is associated with prolonged hospital length of stay. Variables such as donor age and recipient life support status seemed to be predictors of mortality risk among this specific patient population. Addressing these factors, among other high‐risk factors, through improved donor selection and recipient management strategies could possibly enhance the transplant outcomes of patients with marginal grafts. Future research should focus on larger prospective studies to further elucidate these relationships and refine the transplant protocols.

## CONFLICT OF INTEREST STATEMENT

The authors declare no conflicts of interest.

## Supporting information


**Supplemental Figure 1:** Survival analysis of pediatric LT recipients with **A)** macrovesicular steatosis <30% and ≥30%, **B)** microvesicular steatosis <30% and ≥30%.

**Supplemental Figure 2:** Likelihood importance of each variable for predicting survival among pediatric LT recipients using gradient boosting decision trees stratified by steatosis type. AUC: 0.975, sensitivity: 97.1%, and specificity: 92.1%.

Supporting information.

Supporting information.
