# Supplementary figures and images for: Pediatric liver transplant outcomes: A comparative analysis of steatotic donor grafts
Source: J Pediatr Gastroenterol Nutr. 2025 Sep 22;81(5):1260–70. doi: 10.1002/jpn3.70213 (PMC12580464; doi:10.1002/jpn3.70213)

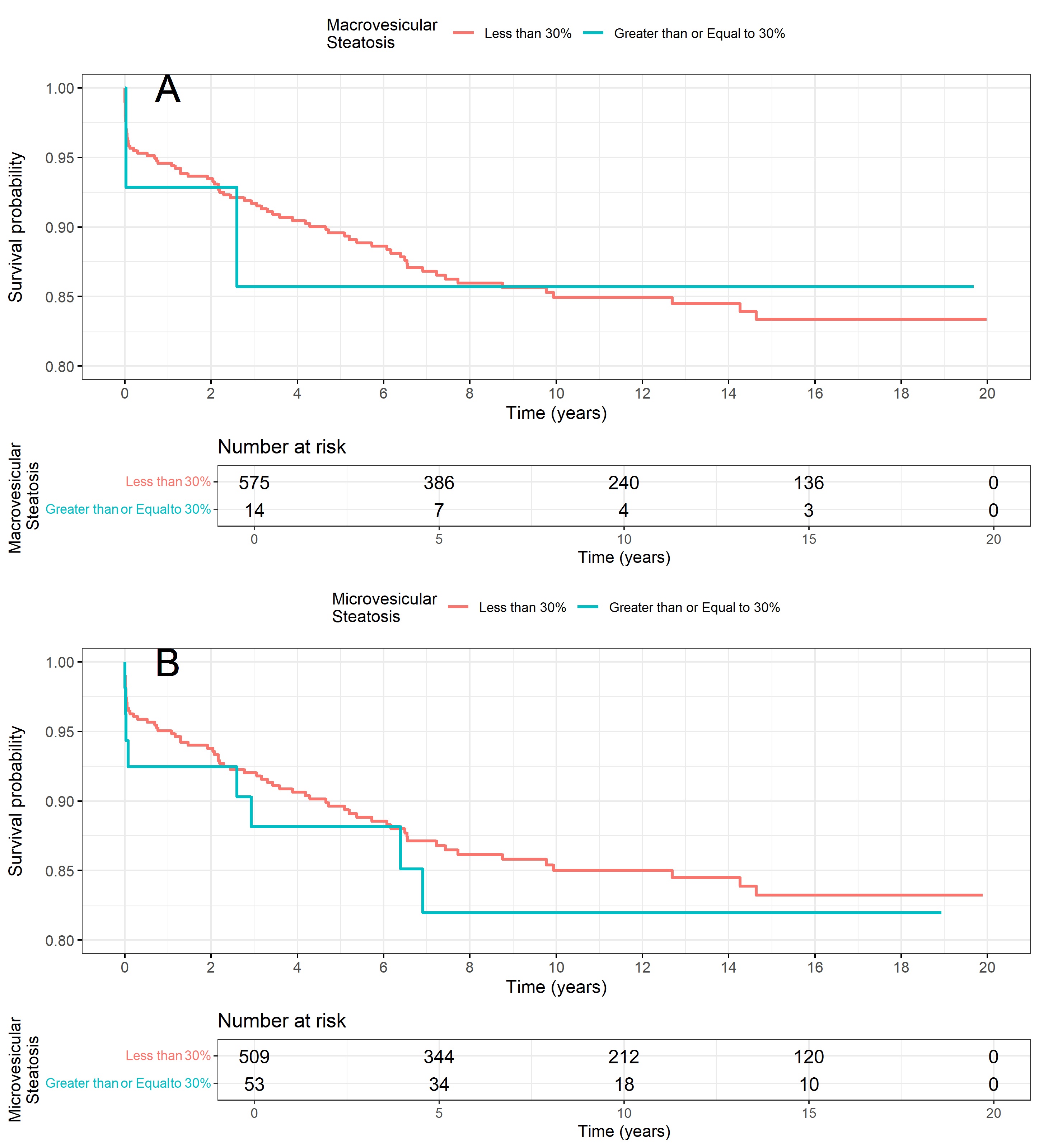

Supplement: Supplementary file 1 — Supplemental Figure 1: Survival analysis of pediatric LT recipients with A) macrovesicular steatosis <30% and ≥30%, B) microvesicular steatosis <30% and ≥30%. [file JPN3-81-1260-s002.jpg]

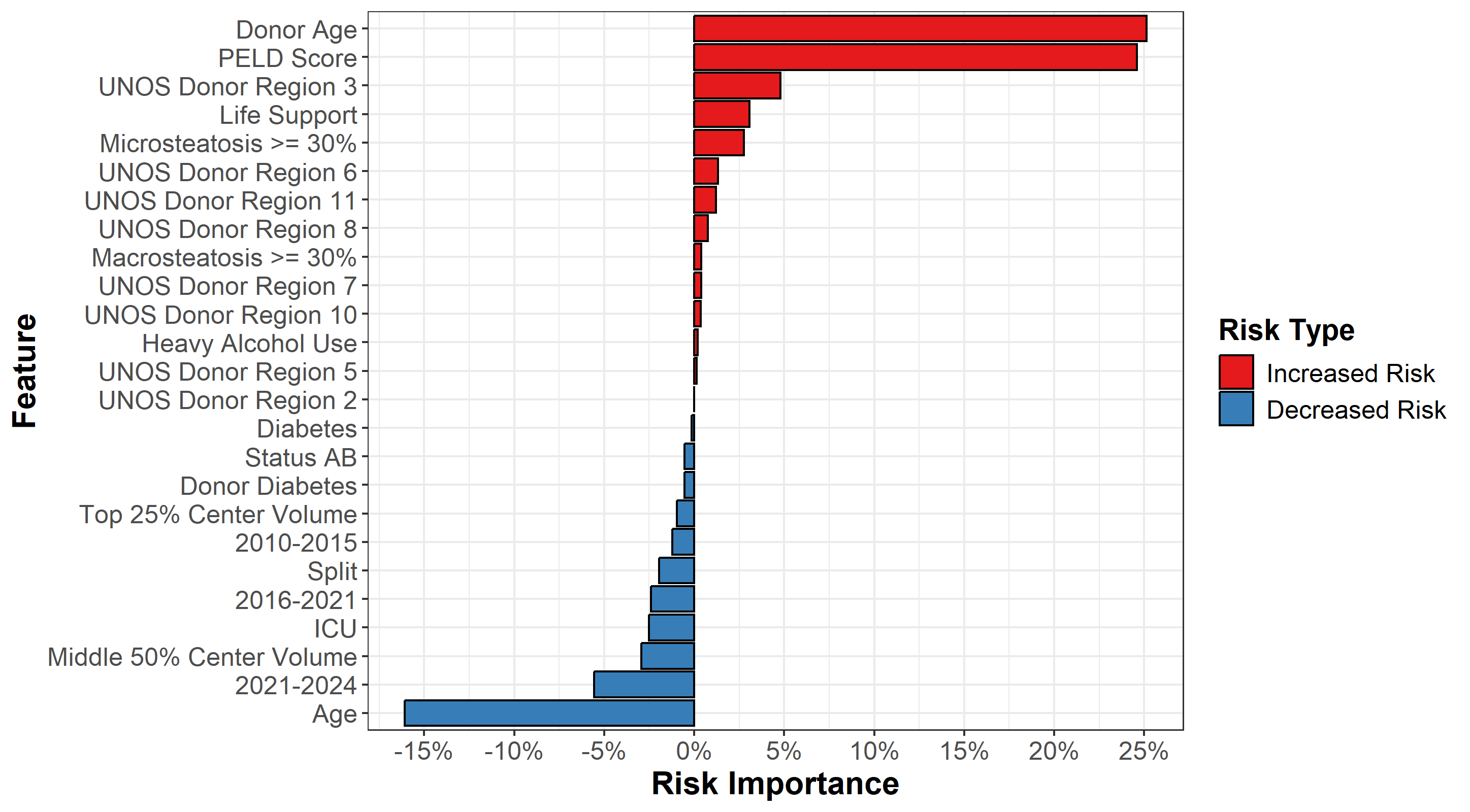

Supplement: Supplementary file 2 — Supplemental Figure 2: Likelihood importance of each variable for predicting survival among pediatric LT recipients using gradient boosting decision trees stratified by steatosis type. AUC: 0.975, sensitivity: 97.1%, and specificity: 92.1%. [file JPN3-81-1260-s003.png]
