# Supplementary material for: Pediatric liver transplant outcomes: A comparative analysis of steatotic donor grafts
Source: J Pediatr Gastroenterol Nutr. 2025 Sep 22;81(5):1260–70. doi: 10.1002/jpn3.70213 (PMC12580464; doi:10.1002/jpn3.70213)
Supplement: Supplementary file 3 — Supporting information. [file JPN3-81-1260-s001.docx]

| **Variable** | **Macrovesicular steatosis<30% (N=575)** | **Macrovesicular steatosis≥30% (N=14)** | **P-value^a^** | **Microvesicular steatosis<30% (N=509)** | **Micro-steatosis≥30% (N=53)** | **P-value^b^** |
| --- | --- | --- | --- | --- | --- | --- |
| **Donor Characteristics** | | | | | | |
| **Age (years)** | 21.16±17.47 | 25.79±16.93 | 0.29 | 21.31±17.7 | 20.49±15.82 | 0.94 |
| **Female Gender** | 261 (45.4%) | 6 (42.9%) | 0.99 | 232 (45.6%) | 24 (45.3%) | 0.99 |
| **Ethnicity** |  |  | 0.56 |  |  | 0.22 |
| - **Non-Hispanic White** | 349 (60.7%) | 8 (57.2%) |  | 321 (63.1%) | 26 (49.1%) |  |
| - **Non-Hispanic Black** | 115 (20.0%) | 1 (7.1%) |  | 101 (19.8%) | 11 (20.8%) |  |
| - **Hispanic** | 92 (16.0%) | 5 (35.7%) |  | 71 (13.9%) | 14 (26.4%) |  |
| - **Asian** | 11 (1.9%) | 0 (0%) |  | 9 (1.8%) | 2 (3.8%) |  |
| - **American Indian/Alaskan Native** | 5 (0.9%) | 0 (0%) |  | 5 (1.0%) | 0 (0%) |  |
| - **Native Hawaiian/Other Pacific Islander** | 1 (0.2%) | 0 (0%) |  | 1 (0.2%) | 0 (0%) |  |
| - **Multiracial** | 2 (0.3%) | 0 (0%) |  | 1 (0.2%) | 0 (0%) |  |
| **Diabetes History** | 25 (4.3%) | 1 (7.1%) | 0.63 | 28 (5.5%) | 0 (0%) | 0.55 |
| **Hypertension History** | 70 (12.2%) | 2 (14.3%) | 0.96 | 65 (12.8%) | 5 (9.4%) | 0.01 |
| **Heavy Alcohol Use History (>2 drinks/day)** | 45 (7.8%) | 0 (0%) | 0.52 | 39 (7.7%) | 5 (9.4%) | 0.74 |
| **History of HCV** | 2 (0.3%) | 0 (0%) | 0.95 | 2 (0.4%) | 0 (0%) | 0.84 |
| **History of HIV** | 0 (0%) | 0 (0%) | 0.99 | 0 (0%) | 0 (0%) | 0.99 |
| **Weight (KG)** | 52.42±29.41 | 67.71±22.93 | 0.07 | 51.87±29.48 | 55.78±29.04 | 0.42 |
| **Body mass index (kg/m^2^)** | 22.45±6.03 | 25.19±5.63 | 0.06 | 22.24±5.99 | 23.41±6.23 | 0.19 |
| **Last creatinine** | 1.24±1.48 | 1.43±1.34 | 0.43 | 1.22±1.44 | 1.45±1.89 | 0.21 |
| **Last total bilirubin** | 0.9±0.88 | 1.31±1.63 | 0.55 | 0.89±0.87 | 1.11±1.12 | 0.14 |
| **Last AST** | 109.51±154.6 | 55.71±37.46 | 0.26 | 107.74±157.47 | 106.62±123.08 | 0.39 |
| **Last ALT** | 93.53±171.58 | 76.29±126.1 | 0.80 | 96.45±180.9 | 64.69±56.41 | 0.90 |
| **Liver Biopsy** | 575 (100%) | 14 (100%) | <0.001 | 509 (100%) | 53 (100%) | <0.001 |
| **Liver Fibrosis** |  |  | 0.06 |  |  | 0.08 |
| - **Fibrosis (unspecified degree)** | 24 (4.2%) | 2 (14.3%) |  | 20 (3.9%) | 6 (11.3%) |  |
| - **Cirrhosis (incomplete and complete)** | 0 (0%) | 0 (0%) |  | 0 (0%) | 0 (0%) |  |
| **Deceased Cardiac Donation** | 3 (0.5%) | 1 (7.1%) | 0.18 | 3 (0.6%) | 1 (<0.1%) | 0.83 |
| **Blood type:** |  |  | 0.82 |  |  | 0.96 |
| - **A** | 168 (29.2%) | 4 (28.6%) |  | 147 (28.9%) | 18 (34.0%) |  |
| - **B** | 51 (8.9%) | 0 (0%) |  | 46 (9.0%) | 5 (9.4%) |  |
| - **O** | 349 (60.7%) | 10 (71.4%) |  | 309 (60.7%) | 30 (56.6%) |  |
| - **AB** | 2 (0.3%) | 0 (0%) |  | 7 (1.4%) | 0 (0%) |  |
| **UNOS Region** |  |  | 0.07 |  |  | 0.01 |
| - **1** | 18 (3.1%) | 0 (0%) |  | 15 (2.9%) | 3 (5.7%) |  |
| - **2** | 39 (6.8%) | 1 (7.1%) |  | 29 (5.7%) | 9 (17.0%) |  |
| - **3** | 132 (23.0%) | 4 (28.6%) |  | 119 (23.4%) | 15 (28.3%) |  |
| - **4** | 28 (4.9%) | 0 (0%) |  | 22 (4.3%) | 5 (9.4%) |  |
| - **5** | 64 (11.1%) | 3 (21.4%) |  | 57 (11.2%) | 4 (7.5%) |  |
| - **6** | 27 (4.7%) | 0 (0%) |  | 26 (5.1%) | 0 (0%) |  |
| - **7** | 65 (11.3%) | 0 (0%) |  | 59 (11.6%) | 4 (7.5%) |  |
| - **8** | 72 (12.5%) | 2 (14.3%) |  | 66 (13.0%) | 0 (0%) |  |
| - **9** | 20 (3.5%) | 3 (21.4%) |  | 21 (4.1%) | 0 (0%) |  |
| - **10** | 41 (7.1%) | 0 (0%) |  | 37 (7.3%) | 1 (1.9%) |  |
| - **11** | 69 (12.0%) | 1 (7.1%) |  | 58 (11.4%) | 9 (17.0%) |  |
| Microvesicular steatosis≥30% | 47 (8.2%) | 5 (35.7%) | 0.002 | 0 (0%) | 0 (0%) | 0 |
| **Macrovesicular steatosis≥30%** | 0 (0%) | 0 (0%) | 0 (0%) | 7 (1.4%) | 5 (9.4%) | <0.001 |
| **Recipient Characteristics** | | | | | | |
| **Age (years)** | 8.13±6.83 | 13.14±4.62 | <0.001 | 8.11±6.8 | 9.28±6.87 | 0.31 |
| **Cold ischemia time (hours)** | 7.06±2.84 | 6.89±1.85 | 0.93 | 7.07±2.93 | 6.97±2.24 | 0.89 |
| **Transplant Type** |  |  | 0.22 |  |  | 0.59 |
| - **Split** | 154 (26.8%) | 1 (7.1%) |  | 375 (73.7%) | 41 (77.4%) |  |
| - **Whole** | 421 (73.2%) | 13 (92.9%) |  | 134 (26.3%) | 12 (22.6%) |  |
| **Ethnicity** |  |  | 0.89 |  |  | 0.96 |
| - **Non-Hispanic White** | 291 (50.6%) | 7 (50.0%) |  | 264 (51.9%) | 25 (47.2%) |  |
| - **Non-Hispanic Black** | 115 (20.0%) | 2 (14.3%) |  | 99 (19.4%) | 12 (22.6%) |  |
| - **Hispanic** | 118 (20.5%) | 5 (35.7%) |  | 102 (20.0%) | 11 (20.8%) |  |
| - **Asian** | 34 (5.9%) | 0 (0%) |  | 30 (5.9%) | 4 (7.5%) |  |
| - **American Indian/Alaskan Native** | 6 (1.0%) | 0 (0%) |  | 5 (1.0%) | 1 (1.9%) |  |
| - **Native Hawaiian/Other Pacific Islander** | 1 (0.2%) | 0 (0%) |  | 1 (0.2%) | 0 (0%) |  |
| - **Multiracial** | 9 (1.6%) | 0 (0%) |  | 7 (1.4%) | 0 (0%) |  |
| - **Unknown** | 1 (0.2%) | 0 (0%) |  | 1 (0.2%) | 0 (0%) |  |
| **Female Gender** | 290 (50.4%) | 4 (28.6%) | 0.18 | 256 (50.3%) | 28 (52.8%) | 0.84 |
| **Diabetes** | 12 (2.1%) | 0 (0%) | 0.99 | 9 (1.8%) | 2 (3.8%) | 0.13 |
| **Body mass index (kg/m^2^)** | 19.42±4.48 | 21.44±4.62 | 0.049 | 19.51±4.56 | 18.96±3.76 | 0.71 |
| **Weight (Kg)** | 33.78±26.18 | 53.85±19.98 | <0.001 | 33.87±26.31 | 36.54±24.68 | 0.48 |
| **Total Bilirubin (mg/dL)** | 11.33±11.93 | 8.41±12.59 | 0.43 | 11.04±11.75 | 13.41±13.42 | 0.13 |
| **Albumin** | 3.2±0.75 | 3.13±0.66 | 0.79 | 3.19±0.76 | 3.24±0.65 | 0.56 |
| **Serum Creatinine (mg/dL)** | 0.7±1.01 | 0.84±0.7 | 0.049 | 0.69±1.02 | 0.87±0.82 | 0.08 |
| **Length of stay (days)** | 28.9±40.85 | 25.64±39.96 | 0.14 | 27.33±37.99 | 42.90±55.14 | 0.01 |
| **Days on Waitlist** | 115.9±244.78 | 106±217.25 | 0.78 | 114.93±233.64 | 149.32±371.50 | 0.32 |
| **UNOS Region** |  |  | 0.002 |  |  | 0.28 |
| - **1** | 23 (4%) | 0 (0%) |  | 21 (4.1%) | 3 (5.7%) |  |
| - **2** | 54 (9.4%) | 0 (0%) |  | 45 (8.8%) | 9 (17.0%) |  |
| - **3** | 113 (19.7%) | 3 (21.4%) |  | 108 (21.2%) | 9 (17.0%) |  |
| - **4** | 29 (5.0%) | 1 (7.1%) |  | 25 (4.9%) | 3 (5.7%) |  |
| - **5** | 57 (9.9%) | 2 (14.3%) |  | 48 (9.4%) | 4 (7.5%) |  |
| - **6** | 22 (3.8%) | 0 (0%) |  | 21 (4.1%) | 0 (0%) |  |
| - **7** | 70 (12.2%) | 0 (0%) |  | 62 (12.2%) | 6 (11.3%) |  |
| - **8** | 83 (14.4%) | 2 (14.3%) |  | 76 (14.9%) | 3 (5.7%) |  |
| - **9** | 29 (5.0%) | 5 (35.7%) |  | 26 (5.1%) | 5 (9.4%) |  |
| - **10** | 32 (5.6%) | 1 (7.1%) |  | 24 (4.7%) | 4 (7.5%) |  |
| - **11** | 63 (11.0%) | 0 (0%) |  | 53 (10.4%) | 7 (13.2%) |  |
| **Education Level:** |  |  | 0.31 |  |  | 0.049 |
| - **None** | 7 (1.2%) | 0 (0%) |  | 7 (1.4%) | 0 (0%) |  |
| - **Grade/Highschool** | 283 (49.2%) | 11 (78.6%) |  | 252 (49.5%) | 28 (52.8%) |  |
| - **College/Bachelor** | 0 (0%) | 0 (0%) |  | 9 (1.8%) | 1 (1.9%) |  |
| - **Postgraduate** | 0 (0%) | 0 (0%) |  | 0 (0%) | 0 (0%) |  |
| **Insurance** |  |  | 0.50 |  |  | 0.84 |
| - **Private** | 262 (45.6%) | 7 (50.0%) |  | 238 (46.8%) | 23 (43.4%) |  |
| - **Medicaid** | 249 (43.3%) | 4 (28.6%) |  | 213 (41.8%) | 26 (49.1%) |  |
| - **Medicare/CHIP** | 30 (5.2%) | 2 (14.3%) |  | 28 (5.5%) | 1 (1.9%) |  |
| - **VA/Other Gov.** | 14 (2.4%) | 1 (7.1%) |  | 12 (2.4%) | 2 (3.8%) |  |
| - **Self/Donation** | 8 (1.4%) | 0 (0%) |  | 7 (1.4%) | 0 (0%) |  |
| - **Foreign** | 11 (1.9%) | 0 (0%) |  | 10 (2.0%) | 1 (1.9%) |  |
| **Life support** | 118 (20.5%) | 2 (14.3%) | 0.81 | 101 (19.8%) | 12 (22.6%) | 0.77 |
| **Portal vein thrombosis** | 33 (5.7%) | 0 (0%) | 0.62 | 23 (4.5%) | 5 (9.4%) | 0.26 |
| **Intensive care unit** | 168 (29.2%) | 3 (21.4%) | 0.77 | 147 (28.9%) | 19 (35.8%) | 0.56 |
| **Ascites** | 226 (39.3%) | 5 (35.7%) | 0.44 | 200 (39.3%) | 22 (41.5%) | 0.90 |
| **Hepatic Encephalopathy** | 188 (32.7%) | 5 (35.7%) | 0.81 | 164 (32.2%) | 23 (43.4%) | 0.19 |
| **Hepatocellular carcinoma** | 4 (0.7%) | 0 (0%) | 0.99 | 25 (4.9%) | 3 (5.7%) | 0.89 |
| **Dialysis** | 51 (8.9%) | 2 (14.3%) | 0.85 | 44 (8.6%) | 6 (11.3%) | 0.68 |
| **MELD/PELD Score at Transplant** | 17.94±14.52 | 18.07±14.22 | 0.87 | 17.71±14.5 | 20.2±13.77 | 0.21 |
| **MELD Exception** | 210 (36.5%) | 5 (35.7%) | 0.054 | 187 (36.7%) | 16 (30.2%) | 0.39 |
| **Status 1A/B** | 210 (36.5%) | 4 (28.6%) | 0.74 | 186 (36.5%) | 19 (35.8%) | 1.0 |
| **Center Volume:** |  |  | 0.96 |  |  | 0.10 |
| - **Lower volume** | 309 (53.7%) | 8 (57.1%) |  | 274 (53.8%) | 28 (52.8%) |  |
| - **Medium volume** | 185 (32.2%) | 4 (28.6%) |  | 159 (31.2%) | 22 (41.5%) |  |
| - **High volume** | 81 (14.1%) | 2 (14.3%) |  | 76 (14.9%) | 3 (5.7%) |  |
| **Transplant Era:** |  |  | 0.10 |  |  | 0.42 |
| - **2004-2009** | 187 (32.5%) | 3 (21.4%) |  | 168 (33.0%) | 12 (22.6%) |  |
| - **2010-2015** | 151 (26.3%) | 3 (21.4%) |  | 132 (25.9%) | 16 (30.2%) |  |
| - **2016-08/2021** | 163 (28.3%) | 8 (57.1%) |  | 146 (28.7%) | 19 (35.8%) |  |
| - **09/2021-2024** | 74 (12.9%) | 0 (0%) |  | 63 (12.4%) | 6 (11.3%) |  |

**Supplemental Table 1**: All donor and recipient characteristics from 2004-2024 stratified by macrovesicular steatosis and microvesicular steatosis <30% and ≥30% and age≤18 years old.

^a^: Comparison between donor grafts with macro-vesicular steatosis < 30% and ≥ 30%

^b^: Comparison between donor grafts with micro-vesicular steatosis < 30% and ≥ 30%

^*^ PELD: Pediatric end stage of liver disease score, UNOS: United Network of Organ Sharing
