# Supplementary material for: Pediatric liver transplant outcomes: A comparative analysis of steatotic donor grafts
Source: J Pediatr Gastroenterol Nutr. 2025 Sep 22;81(5):1260–70. doi: 10.1002/jpn3.70213 (PMC12580464; doi:10.1002/jpn3.70213)
Supplement: Supplementary file 4 — Supporting information. [file JPN3-81-1260-s004.docx]

| **Variables** | **Likelihood (%)^1^** |
| --- | --- |
| **Donor age** | +25.1% |
| **Donor macro-steatosis** | +0.4% |
| **Donor micro-steatosis** | +2.8% |
| **Donor diabetes history** | +0.5% |
| **Transplant Type (Split)** | -2.0% |
| **Recipient life support requirements** | +3.1% |
| **PELD score** | +24.6% |
| **Recipient age** | +16.1% |
| **Recipient ICU stay** | +2.5% |
| **Recipient diabetes** | +0.1% |
| **Transplant era (ref: 2004-2009)** |  |
| - **2010-2015** | -1.2% |
| - **2016-08/2-21** | -2.4% |
| - **09/2021-2024** | -5.6% |
| **Center volume (ref: low volume)** |  |
| - **Middle volume** | -2.9% |
| - **High volume** | -1.0% |
| **UNOS Region (Ref: Region 1)** |  |
| - **2** | +0.003% |
| - **3** | +4.8% |
| - **4** | Dropped from Lasso |
| - **5** | +0.1% |
| - **6** | +1.3% |
| - **7** | +0.4% |
| - **8** | +0.8% |
| - **9** | Dropped from Lasso |
| - **10** | +0.4% |
| - **11** | +1.2% |

**Supplemental Table 2:** Relative importance for predicting mortality outcomes of study recipients using gradient boosting decision trees.

Model: AUC: 0.975, sensitivity: 97.1%, and specificity: 92.1%.

^1^: +: increased association with the relative importance in detecting likelihood of mortality, -: decreased association with the relative importance in detecting likelihood of mortality

^*^ PELD: Pediatric end stage of liver disease score, UNOS: United Network of Organ Sharing, AUC: Area under the curve, Region 1: Connecticut, Maine, Massachusetts, New Hampshire, Rhode Island, Eastern Vermont; Region 2: Delaware, District of Columbia, Maryland, New Jersey, Pennsylvania, West Virginia, Northern Virginia; Region 3: Alabama, Arkansas, Florida, Georgia, Louisiana, Mississippi, Puerto Rico; Region 4: Oklahoma, Texas; Region 5: Arizona, California, Nevada, New Mexico, Utah; Region 6: Alaska, Hawaii, Idaho, Montana, Oregon, Washington; Region 7: Illinois, Minnesota, North Dakota, South Dakota, Wisconsin; Region 8: Colorado, Iowa, Kansas, Missouri, Nebraska, Wyoming; Region 9: New York, Western Vermont; Region 10: Indiana, Michigan, Ohio; Region 11: Kentucky, North Carolina, South Carolina, Tennessee, Virginia
